# Surgical Excision with Forehead Flap as Single Modality Treatment for Basal Cell Cancer of Central Face: Single Institutional Experience of 50 Cases

**DOI:** 10.1155/2014/320792

**Published:** 2014-01-28

**Authors:** Jagdeep Rao, Harsh Deora

**Affiliations:** ^1^Department of Plastic Surgery, Sawai Man Singh Medical College, Jaipur, Rajasthan 300204, India; ^2^Department of General Surgery, Sawai Man Singh Medical College, Jaipur, Rajasthan 300204, India

## Abstract

Basal cell carcinoma (BCC) is the most common skin cancer worldwide. The WHO has defined it as “a locally invasive, slowly spreading tumor which rarely metastasizes, arising in the epidermis or hair follicles and in which the peripheral cells usually simulate the basal cells of the epidermis.” Here we discuss the management of BCCs of central face with surgical excision and reconstruction with forehead flap as single modality treatment. *Material and Methods*. This is a retrospective review of 50 patients who underwent surgical excision of BCC involving the facial region followed by primary reconstruction using forehead flaps at a single institution. There were 20 males and 30 females, mean age of 59 years. *Results*. No recurrence at primary site was observed during the follow-up of 1–4 yrs. There was no ectropion or exposure sequela. However, epiphora was evident. Size of lesions ranged from 2 to 6 cm. Keloid formation was seen in 2 (4%) patients. Functional and cosmetic outcomes were satisfactory. *Conclusion*. For the face, the best reconstructive effort eventually fails in the face of tumor recurrence. The forehead flap represents one of the best methods for repair of extensive facial defects. Complete tumor extirpation, the primary event, is the key.

## 1. Introduction

Basal cell carcinoma (BCC) is the most common skin cancer worldwide [1]. The World Health Organization Committee defined it based on the histological typing of skin tumors as “a locally invasive, slowly spreading tumor which rarely metastasizes, arising in the epidermis or hair follicles and in which, in particular, the peripheral cells usually simulate the basal cells of the epidermis” [2]. BCC constitutes approximately 75% of nonmelanoma skin cancers. It is usually observed in older patients, especially in those frequently and intensively exposed to ultraviolet radiation during their lives. The most typical site of BCC is uncovered skin directly exposed to the sun. Thus, BCC is often observed in head and neck areas, especially the eyelid and nose. It occurs chiefly in the elderly and is more common in males. Generally speaking, the tumor grows slowly and behaves in a nonaggressive fashion. BCC may be treated by surgery, cryotherapy, radiotherapy, and curettage and electrodessication [3]. Other less frequently used treatment modalities include the topical application of 5-fluorouracil (5-FU) ointment, laser treatment, and systemic chemotherapy [4]. To achieve a favorable outcome, it is important to recognize the histological subtypes, identify the anatomic locations that can increase the risk of spread, and understand the limitations of all available treatment modalities. If surgical defects are repaired, it is necessary to carefully plan the reconstruction after the tumor margins have been cleared. The forehead flap is an axial flap based on supraorbital/supratrochlear blood vessels. The flap has been used extensively in nasal reconstruction [5]. This paper discusses the management of basal cell carcinomas of the facial region with surgical excision and reconstruction with forehead flap as single modality treatment.

## 2. Patients and Methods

This is a retrospective review of 50 patients who underwent surgical excision of BCC involving the facial region followed by primary reconstruction using forehead flaps. Patients who underwent reconstruction without forehead flaps were excluded. There were a total of 50 patients treated over a period of 3 years (2009–2012). There were 20 males and 30 females with a mean age of 59 years (range 50–71 years). In all patients, the diagnosis of BCC was confirmed by an incisional biopsy prior to definitive management. None of the patients had evidence of regional or distant metastasis (Table 1). This work was carried out in the Department of Plastic Surgery, Sawai Man Singh Medical College, Jaipur. The duration of follow-up ranged from 1 to 4 yrs.

### 2.1. Surgical Procedure

The forehead flap is a 2-stage procedure, and patients should receive preoperative counselling concerning their appearance between the first and second stages of the procedure. Thorough preoperative planning, including assessment of the defect, hairline height, and forehead laxity, is important. Patients should be given wound care instructions and realistic goals about the final outcome of their reconstruction. The lesion gross outline was marked by dots, and then about 5 mm free healthy margin was marked by a continuous line. The proposed reconstruction flap was marked at the same time. The defects following excision ranged from 3 × 4 cm to 5 × 7 cm in size (Table 2). In all cases, excision of the lesion included periosteum and, hence, bare bone was evident at the base of the defects.

### 2.2. Surgical Reconstruction

**Figure 1:**
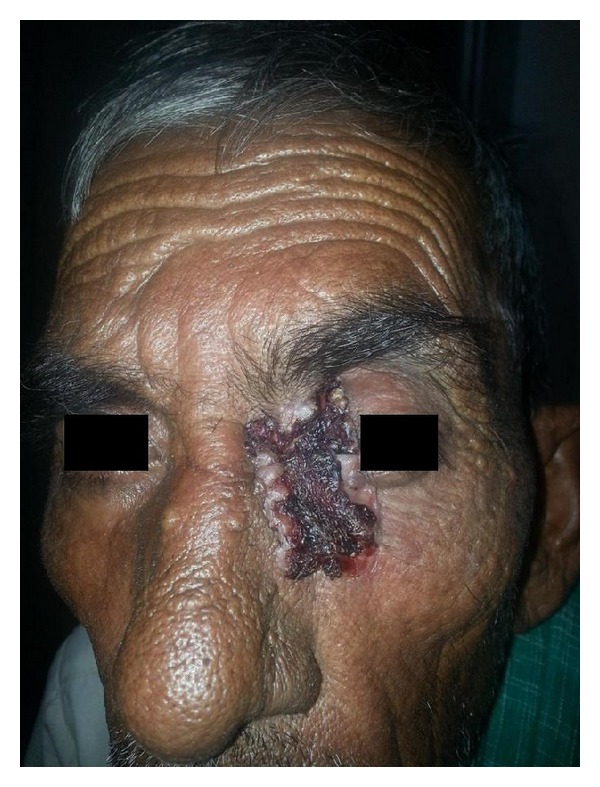
Preoperative image of BCC face involving medial canthus.

**Figure 2:**
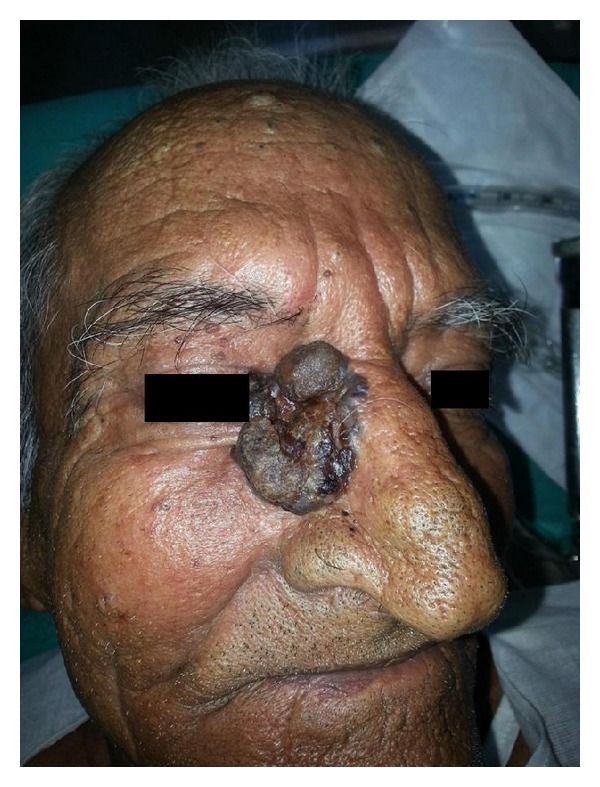
Preoperative image of BCC face.

In all cases, the median or paramedian pedicled forehead flap was the main method of surgical reconstruction. For difficult areas such as those involving the medial canthus or the eyelids, the flap was rotated toward the defect for repair of the medial canthus, anterior lamella of the lower eyelid, and the side of the nose. Mucosal or skin grafts were sutured to the undersurface of the flap to reconstruct the conjunctiva. No cartilage grafts were used (to reconstruct the tarsal plate) because the flaps were “stiff” enough to provide self-support. Whenever possible (especially with defects extending to the nasolabial fold), primary closure of the edge of the defect was done. Lacrimal system reconstruction was not performed in any of the patients. The donor sites of the forehead flaps were covered using split thickness skin grafts in 20 patients and primary closure using skin advancement in the remaining 30 patients. Forehead flaps were divided 3 weeks later. The pedicles were returned back to their donor sites in the forehead after excision of the skin grafts. Demonstrative examples are shown (Figures 1 and 2).

Patients are discharged home after surgery or kept overnight for observation and wound care. It has been our experience that most patients who undergo extensive nasal reconstruction appreciate an overnight admission to aid in wound care and to ensure adequate intravenous hydration. Prescriptions at discharge include a broad-spectrum antibiotic, which is to be taken for 7 to 10 days. A mild pain reliever, such as diclofenac sodium, and an antiemetic are often prescribed. Postoperative wound care consists of twice daily cleansing of the suture lines with spirit and application of an antibiotic ointment or petroleum jelly.

## 3. Results

**Figure 3:**
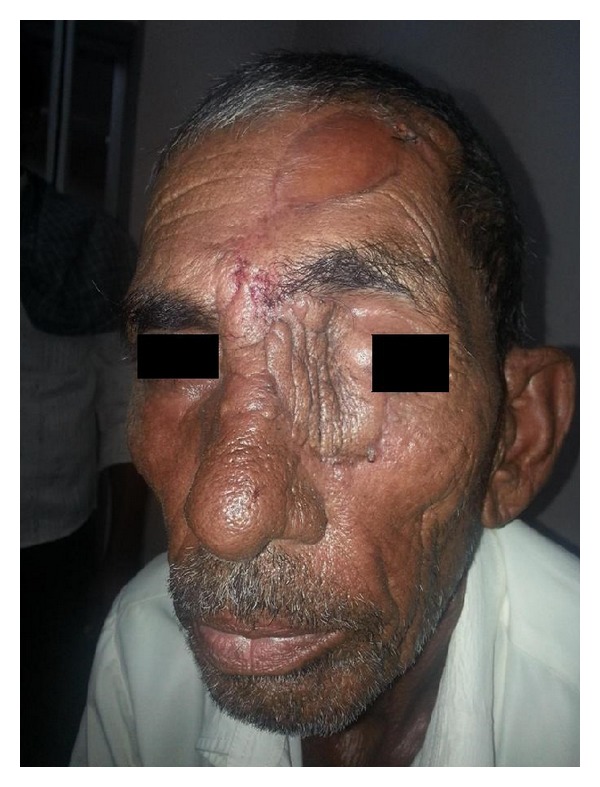
Reconstruction using forehead flap with SSG closure.

**Figure 4:**
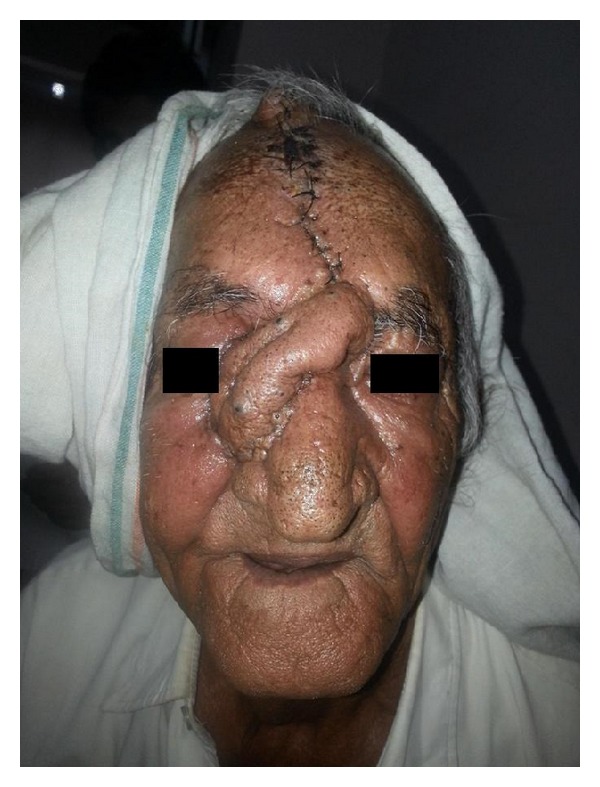
Reconstruction using forehead flap with primary closure.

All patients tolerated the surgical procedures well with no systemic or anesthesia-related complications. There were no infections or hematomas. All flaps survived completely and there were no instances of skin/mucosal graft loss (Figures 3 and 4).

Follow-up ranged from 1 to 4 years (mean of 3 years). Tumor recurrence was not seen in any of the patients, during this period. Functionally, whenever the eyelid was involved, there was no ectropion and the margin was well aligned and stable. Eyelid closure was adequate and there were no exposure sequelae. However, epiphora was evident since lacrimal system reconstruction was not performed.

**Figure 5:**
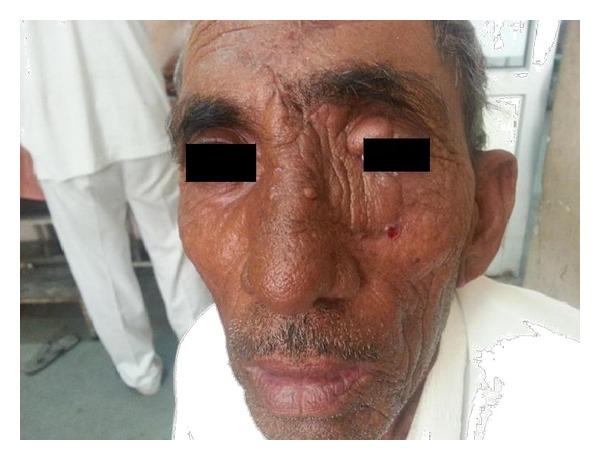
Final result at follow-up.

Cosmetically, there were some color mismatch and no eyelashes. All patients required debulking of the flaps because of the bulky appearance. Debulking was done 3–6 months following the reconstructive procedures. In 30 patients, the entire forehead donor site was closed at the time of primary reconstruction. In the rest of the 20 patients, partial graft loss was observed in 5 of them which was later excised at the time of dressing and was allowed to heal secondarily. Keloid formation was seen in 2 patients (4%). All patients were satisfied with the functional and cosmetic outcomes (Figure 5).

## 4. Discussion

Basal cell carcinoma grows slowly and is painless. A lesion that bleeds easily or does not heal well may be suspected for BCC. The majority of these cancers occur on areas of skin that are regularly exposed to sunlight or other ultraviolet radiation as in the midface [6]. They may also appear on the scalp. All managed cases gave in the history long duration of exposure to the sunlight due to either their occupations (farmers) or social hobbies (swimming for long duration in summer vacations). All cases had lesions on the midface, where exposure to sun is intense.

Basal cell skin cancer used to be more common in people over age of 40 [7]. In our series all cases (100%) were senile and over age of 50 years, as long period and repeated exposure to sunlight are required to introduce the malignant changes in the skin.

Basal cell skin cancers almost never spread, but metastatic cases have been reported. If left untreated, it may grow into surrounding areas and destroy nearby tissues and bone [8, 9]. We have not recorded metastases in our cases. Multiple lesions on the face are common, and new lesions may appear during various years of follow-up, as the predisposing factors cause wide and diffuse skin changes which are not localized to one site [10]. In our series, no patient had multiple lesions on the face on initial presentation. The rate of recurrence is reported to vary and between 6 and 10% [11]. In this study, in the duration of follow-up, no patient developed recurrence at sites of previous resections. Excision of large facial malignant ulcer with forehead flap based on the supratrochlear artery or the frontal branch of a side superficial temporal artery in a 1-stage operation [12] has also been reported but the series is usually small and a two-stage reconstruction offers a better uptake, especially in older patients.

The criterion for surgical treatment varies depending on the size, depth, and location of the lesions. The aim was to excise the tumor radically with gross 5 mm free margins and to reconstruct the defect with the least cosmetic deformity, taking into consideration putting the line of resection in or parallel to normal skin creases.

Periocular reconstruction following the excision of cutaneous malignancy includes providing stable eyelid margin, providing reasonable symmetry with smooth internal surfaces, providing adequate eyelid closure to avoid exposure sequelae, restoring normal tension, and providing sufficient horizontal and vertical eyelid dimensions for maximal function [13, 14]. Although various local flaps have been used for the reconstruction of medial canthus/adjacent eyelid defects [15, 16], we find the forehead flap to be the most suited for these defects. In fact, we believe it should be considered as the flap of choice in large defects mainly because it satisfies the principles of periocular reconstruction. The proximity of the flap and the arc of its rotation make it easier to provide a stable eyelid margin. The flap is an axial type with rich blood supply and, hence, it could be of sufficient size to ensure adequate reconstruction of large defects without tension or vertical eyelid deficiency. The rich blood supply at the distal end of the flap also ensures good take of the mucosal or skin grafts which provide conjunctival reconstruction in full thickness eyelid defects.

For nasal reconstructions, the midline forehead skin flap can serve as a cover for any nasal reconstruction from severe tip and ala loss to a total nasal defect. Using this flap, aesthetic and functional reconstruction can be achieved by creating a nose that blends well with the face. The seagull-shaped flap is based on one of the supratrochlear vessel bundles. Its vertical axis is placed over the midline of the forehead, and the wings are designed to lie in natural transverse creases. The forehead flap is elevated and transposed 180° to cover the new nose. The body of the seagull lies along the bridge, the wings curl at the ala and turn into the nostril sills, and the seagull head and neck create the tip and columella [17].

Despite all these advantages, some disadvantages when using forehead flaps should be mentioned, which include the two-stage procedure, the color mismatch, the bulkiness of the flap, and the donor site scar.

There are also few rare complications such as sepsis and necrosis which are avoided by gentle care of flaps, antibiotics, plenty of fluid intake, wide pedicle covered with petroleum gauze, and avoidance of excess torsion at base [18].

## 5. Conclusion

The face is one of the most common locations for skin cancer and frequently represents a significant challenge for reconstruction after surgical excision. Reconstruction of defects created by removal of cancer represents the secondary event in successful skin cancer treatment. Complete tumor extirpation, the primary event, is the key. The best reconstructive effort eventually fails in the face of tumor recurrence. The forehead flap represents one of the best methods for repair of extensive facial defects. Outstanding functional and cosmetic results can be achieved. Proper execution requires considerable technical skill and experience. Preoperative counseling is vitally important. Also, a thorough understanding of anatomy and aesthetics is required.

## Figures and Tables

**Table 1:** Distribution of lesions on face.

Malar region	15
Nose	13
Eyelids	7
Cheeks	7
Forehead	4
Lips	2
Others	2

Total	50

**Table 2:** Size of lesions.

2-3 cm	28
3-4 cm	15
4-5 cm	5
5-6 cm	2
